# Traditional and individual care pathways in gender-affirming healthcare for transgender and gender-diverse individuals - results from the ENIGI follow-up study

**DOI:** 10.1038/s41443-025-01085-8

**Published:** 2025-05-08

**Authors:** Andreas Koehler, Inga Becker-Hebly, Els Elaut, Baudewijntje Kreukels, Peer Briken, Gunter Heylens, Thomas, D. Steensma, Timo Nieder

**Affiliations:** 1 Institute for Sex Research, Sexual Medicine, and Forensic Psychiatry, University Medical Center Hamburg-Eppendorf, Hamburg, Germany; 2 Center of Sexology and Gender, University Hospital Ghent, Ghent, Belgium; 3 Department of Medical Psychology, The Center of Expertise on Gender Dysphoria Amsterdam, VU University Medical Center Amsterdam, Amsterdam, the Netherlands

**Keywords:** Quality of life, Diagnosis

## Abstract

Treatment requests in transgender healthcare are heterogenous and not all transgender and gender-diverse individuals want to undergo the various transition-related medical interventions offered. This study aims to explore demographic and treatment-related predictors associated with different transgender care pathways in a multicenter, multinational clinical setting. In this follow-up study, 539 adult participants from Belgium, Germany, and the Netherlands took part and were categorized as following a ‘traditional’ care pathway (i.e., undergoing all transition-related interventions), an ‘individual’ care pathway (i.e. any course of treatment deviating from the traditional pathway), or ‘no care’ pathway (i.e. not seeking transition-related medical interventions.). We analyzed differences in demographic (e.g., gender identity) and clinical variables (e.g., treatment satisfaction), conducting logistic regression analysis and descriptive subgroup analysis. Participants with a non-binary gender were 6.7 times more likely to follow an individual care pathway, while participants with higher treatment satisfaction were less likely to follow an individual care pathway (Odds Ratio: 0.6). We identified four patterns of individual transgender care pathways, some as a function of the sex assigned at birth. The present study provides valuable insights into demographic and treatment-related predictors associated with different transgender care pathways. Healthcare providers should be aware of individual transgender care pathways and the association with (non)-binary genders to provide tailored transgender healthcare and ensure individualized, high-quality service provision.

## Introduction

**Fig. 1:**
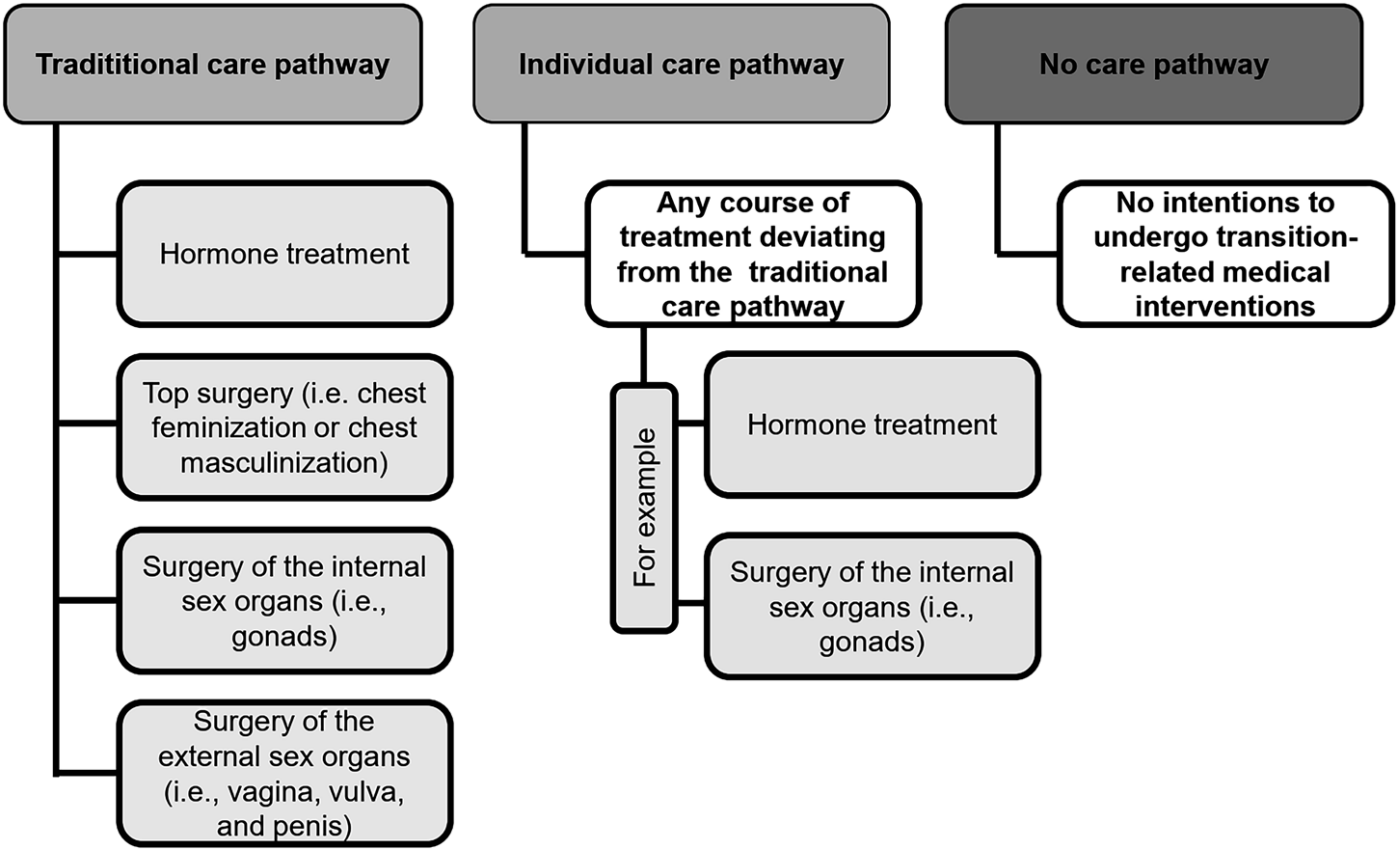
Transgender care pathways.

Transgender and gender-diverse (TGD) individuals experience their gender to be different from the sex they were assigned to at birth. They might identify binary as the ‘opposite’ gender (man, woman) or non-binary. Non-binary individuals experience themselves as moving between male and female (e.g., genderfluid), beyond the gender binary (e.g., genderqueer), or do not identify with any kind of gender at all (e.g., agender) [1]. Additionally, non-binary individuals can include a variety of additional cultural and personal experiences, including identities rooted in specific traditions or languages, such as Two-Spirit in some Indigenous cultures. However, all of these concepts are not mutually exclusive, and people might use multiple terms to describe their identity (e.g., genderqueer trans woman). Gender-affirming care for TGD individuals focuses on medical interventions to support a person’s transition to living according to their gender. This includes mental health counseling (e.g., to cope with minority stress) [2], hormone therapy, gender-affirming surgery (e.g., genital reconstructive surgeries), and additional interventions (e.g., hair removal, speech therapy [3], in the following: transition-related interventions [4]). Transition-related medical interventions contribute to lower gender dysphoria and increase mental health and quality of life in TGD individuals [5–10]. A transgender healthcare pathway is the individual course of various medical interventions a person follows to help them transition into their gender socially and physically. Historically, the expectation of a linear course of treatment, where TGD individuals undergo most or all transition-related interventions, ending with gender-affirming genital surgery, has dominated transgender healthcare [11–14]. However, not all TGD individuals want to follow a ‘traditional’ care pathway [13, 15–17]. Following such individual care pathways, some TGD individuals, for example, only access hormone treatment or single surgical procedures (e.g., mastectomy) but do not desire gender-affirming genital surgery [15] (Fig. 1). Data from the U.S. indicate that overall, about 11% of TGD individuals undergo gender-affirming genital surgery, while 80% of those who underwent some kind of gender-affirming procedure had gender-affirming genital surgery [18]. It is important to notice that undergoing gender-affirming genital surgery is also influenced by how legal gender recognition is regulated in a country. In several European countries, undergoing these procedures was or still is a mandatory requirement for changing one’s name or gender on birth certificates and other official documents [19].

Reasons for not undergoing gender-affirming genital surgery appear to be manifold and include non-binary gender identities [15, 20], a significant lowering of gender dysphoria by other interventions, fear of medical complications [21, 22], limited genital dysphoria [13], and general barriers to transgender healthcare (affordability, lack of insurance coverage, discrimination by healthcare providers) [23–26].

There is still limited research investigating what individual characteristics of TGD individuals and circumstantial factors of transgender healthcare contribute to the variety of individual care pathways. By exploring demographic and treatment-related predictors associated with individual and traditional care pathways, this study aims to contribute to a more comprehensive picture of individual treatment requests of TGD individuals.

## Methods

### Study design

The present analysis is part of a follow-up study carried out within the European Network for the Investigation of Gender Incongruence (ENIGI) [27]. The study was developed as a paper-pencil and web-based survey to investigate adult TGD individuals at three specialized clinics (Amsterdam, Ghent, Hamburg). At baseline, the study’s goal was to include all individuals older than 17 years of age who attended one of the clinics. Therefore, no additional inclusion criteria needed to be defined. At follow-up, the goal was to include all participants who participated at baseline within the study period of interest. Exclusion criteria were the lack of sufficient information regarding treatment or incomplete questionnaire data. TGD individuals who completed the assessment protocol (people who gave informed consent and filled out the ENIGI baseline questionnaires and a series of standardized questionnaires handed out at the initial counseling session) between 2007 and 2009 were contacted between July and September 2013 (follow-up 1). TGD individuals who completed the assessment protocol between 2011 and 2013 were contacted between September 2017 and April 2018 (follow-up 2). The 2010 cohort was excluded to obtain similar follow-up periods. The local ethics committees approved the initial and the follow-up studies (Amsterdam: METc 2008.108 (A2017.342); Ghent: 2013/153; Hamburg: 12/2017-PTK-HH).

### Participants

Participants were invited by phone, e-mail, or regular mail to fill out an online follow-up survey. All participants gave informed consent. A total of 1089 TGD individuals were invited for the study. 550 individuals (response rate: 50.51%) both consented and filled out the survey (Amsterdam, n = 295; Ghent, n = 157; Hamburg, n = 98). Of these 550 participants, 11 participants were excluded because we lacked sufficient information concerning their treatment. Therefore, 539 participants were included in the final analysis.

### Measures

Biographical data (e.g., age) and clinical information (e.g., gender) were assessed by a self-developed questionnaire. The sex assigned at birth was assessed by the clinical records. Participants were asked to indicate their gender (male, female, trans woman, trans men, in between, other) and specify their answer by free text if necessary. Afterward, we categorized gender as binary (woman, trans woman, man, trans man) or non-binary (in between, non-binary gender identity specified in the ‘other’ option). Participants were asked what transition-related medical interventions they had received. Due to inconsistencies and missing information in the self-report data, information on transition-related interventions was also retrieved from clinical records. Inconsistencies were resolved by checking clinical records. All participants were asked if they intended to seek further transition-related interventions. Based on the treatment data, we constructed a polytomous (divided into more than two parts or categories) outcome of belonging to a “traditional transgender care pathway (TCP)”, an “individual transgender care pathway (ICP)”, and a “no transgender care pathway (NCP)”. By being assigned to the TCP group, a participant undergoes or plans to undergo hormone treatment, top surgery (breast augmentation, mastectomy), surgery of the internal sex organs (i.e., gonads), and surgery of the external sex organs (i.e., vagina, vulva, and penis). An ICP is defined as any course deviating from the traditional treatment pathway (e.g., a person who receives hormone therapy and underwent surgery of the internal sex organs, but not of the external sex organs). Participants from the NCP group have or had no intentions of undergoing any transition-related medical interventions (Fig. 1).

Treatment satisfaction and satisfaction with physical appearance were assessed using 5-point Likert scales. If complications occurred, participants were asked to specify these. Traumatic events (e. g. serious physical injuries), chronic health concerns, medication besides hormones, and hospitalization for mental health concerns were asked using yes-no questions. A detailed description of the measures and references to the questionnaires used can be found in the supplementary material.

### Statistical analysis

IBM SPSS Statistics, version 27, was used for statistical analyses. We conducted bivariate analyses across each of the basic demographics and clinical information to examine significant associations with the primary outcome of interest (belonging to one of the transgender care pathways). All statistical comparisons were made between all three groups (TCP, ICP, NCP) and between the ICP and the TCP group separately. *χ*^*2*^ tests were calculated to assess differences in categorical variables. Cramer V was calculated to measure the strength of the relationship. As the data did not fit the assumptions for parametric statistics (i.e., homogeneity of variance), Kruskal-Wallis-tests and Mann-Whitney U-tests were calculated to determine differences between the groups in continuous variables. The Dunn’s-test was used for post-hoc pairwise comparisons. Comparisons between the countries were also calculated and can be found in the supplementary material. To create a prediction model of belonging to one of the care pathways (ICP, TCP), we conducted logistic regression analyses. The NCP group was excluded from the regression analysis due to the small number of cases. We entered all covariates that showed statistical significance in the bivariate analyses. The covariates were entered block-wise in the model (Demographics, treatment-related predictors). In case of missing data, participants were excluded listwise. We bootstrapped confidence intervals of the regression weights based on 5000 samples. Hosmer & Lemeshow, Cox & Snell, and Nagelkerke *R*^*2*^ were used as coefficients of determination. We conducted diagnostics on all relevant variables to check the assumptions for logistic regression models (e.g., independence of errors, incomplete information from the predictors). Cohens f^2^ was calculated as an effect size measure for the logistic regression model. To deal with the problem of multiple comparisons, a Bonferroni correction [28] was adopted with the statistical significance set for a p-value less than 0.002 (0.05 divided by 28).

Finally, we performed a descriptive subgroup analysis of the ICP group, assessing the participants’ different individual treatment pathways and associated demographic factors (sex assigned at birth, non-binary gender).

### Results

The total sample consisted of 539 participants. Two hundred fifty individuals were assigned to the TCP group, 262 to the ICP group, 27 to the NCP group. Demographic characteristics of the sample and differences between the groups are presented in Table 1. Regarding certainty about one’s gender, post-hoc Dunn’s-test found that the NCP group felt less certain about their gender than both the TCP group (z = 167.6, p = 0.000) and the ICP group (z = 166.3, p = 0.000). For satisfaction with physical appearance, post-hoc Dunn’s-test found that only the TCP group and the NCP group differed but did not survive Bonferroni correction (z = 154.4, p = 0.043). The NCP group felt less satisfied with their physical appearance than the TCP group.

**Table 1:** Sample characteristics and differences between the groups.

	Total sample	NCP^a^	TCP^b^	ICP^c^	Statistics (NCP vs. TCP vs. ICP)^d^	Statistics (TCP vs. ICP)^e^
N (%)	539 (100.0)	27 (5.0)	250 (46.4)	262 (58.6)		
Age (Mean, Mdn)	38.70, 36.00	41.52, 38.00	38.23, 35.00	38.87, 35.00	H (2, N = 515) = 1.4; p = 0.489	*U* (*N*_Trad_ = 242, *N*_Ind_ = 248) = 29336.5, *z* = −0.43, *p* = 0.668
Missing	24 (4.5)	2 (7.4)	8 (3.2)	14 (5.3)		
Education						
Low	46 (8.5)	0 (0.0)	23 (9.2)	23 (8.8)	χ^2^ (2, N = 520) = 2.4; p = 0.675	χ^2^ (2, N = 497)= 0.1; p = 0.991
Middle	255 (47.3)	12 (44.4)	121 (48.4)	122 (46.6)		
High	219 (40.6)	11 (40.7)	102 (40.8)	106 (40.5)		
Missing	19 (3.5)	4 (14.8)	4 (1.6)	11 (4.2)		
Employment						
Employed	343 (63.6)	17 (63.0)	168 (67.2)	158 (60.3)	χ^2^ (2, N = 484) = 3.7; p = 0.165	χ^2^ (1, N = 464) = 1.7; p = 0.223
not employed	141 (26.2)	3 (11.1)	62 (24.8)	76 (29.0)		
Missing	55 (10.2)	7 (25.9)	20 (8.0)	28 (10.7)		
Average income (regarding poverty threshold)						
Below	98 (18.2)	3 (11.1)	46 (18.4)	49 (18.7)	χ^2^ (2, N = 518) = 14.8; p = 0.005 (V = 0.120)	χ^2^ (2, N = 495) = 11.3; p = 0.001 (V = 0.170)
Around	114 (21.2)	6 (22.2)	37 (14.8)	71 (27.1)		
Above	306 (56.8)	14 (51.9)	162 (64.8)	130 (49.6)		
Missing	21 (3.9)	4 (14.8)	5 (2.0)	12 (4.6)		
Sex assigned at birth						
Male	322 (59.7)	19 (70.4)	143 (57.2)	160 (61.1)	χ^2^ (2, N = 539) = 2.1; p = 0.355	χ^2^ (1, N = 512) = 0.0.8; p = 0.418
Female	217 (40.3)	8 (29.6)	107 (42.8)	110 (38.9)		
Missing	0 (0.0)	0 (0.0)	0 (0.0)	0 (0.0)		
Non-binary gender						
Yes	46 (8.5)	10 (37.0)	7 (2.8)	29 (11.1)	χ^2^ (2, N = 539) = 40.8; p = 0.000 (V = 0.275)	χ^2^ (1, N = 512) = 13.4; p = 0.000 (V = 0.162)
No	493 (91.5)	17 (63.0)	243 (97.2)	233 (88.9)		
Missing	0 (0.0)	0 (0.0)	0 (0.0)	0 (0.0)		
Certainty about gender identity (Mean, Mdn)	4.53, 5.00	3.31, 4.00	5.59, 5.00	4.53, 5.00	H (2, N = 490) = 25.6; p = 0.000	*U* (*N*_Trad_ = 239, *N*_Ind_ = 238) = 28573.0, *z* = 0.11, *p* = 0.914
Missing	49 (9.1)	14 (51.9)	11 (4.4)	24 (9.2)		
No. transition-related interventions (Mean, Mdn)	2.98, 3.00	–	3.83, 4.00	2.48, 3.00	–	*U* (*N*_Trad_ = 250, *N*_Ind_ = 262) = 12501.0, *z* = −12.53, *p* = 0.000
Missing	0 (0.0)	–	0 (0.0)	0 (0.0)		
Treatment satisfaction (Mean, Mdn)	4.28, 4.40	–	4.42, 4.50	4.13, 4.25	–	*U* (*N*_Trad_ = 216, *N*_Ind_ = 201) = 17494.5, *z* = −3.47, *p* = 0.001
Missing	122 (22.6)	–	34 (13.6)	61 (23.7)		
No. of medical complications (Mean, Mdn)	0.61, 0.00	–	0.76, 0.00	0.53, 0.00	–	*U* (*N*_Trad_ = 250, *N*_Ind_ = 262) = 28970.5, *z* = −2.56, *p* = 0.010
Missing	27 (5.0)	–	0 (0.0)	0 (0.0)		
Satisfaction with physical appearance (Mean, Mdn)	4.00, 4.00	2.75, 3.00	4.11, 4.00	3.91, 4.00	H (2, N = 461) = 9.2; p = 0.010	*U* (*N*_Trad_ = 223, *N*_Ind_ = 234) = 28743.0, *z* = −2.01, *p* = 0.045
Missing	78 (14.5)	23 (85.2)	27 (10.8)	28 (10.7)		
Traumatic events						
Yes	95 (17.6)	4 (14.8)	35 (14.0)	56 (21.4)	χ^2^ (2, N = 520) = 5.5; p = 0.059	χ^2^ (1, N = 497) = 5.4; p = 0.021 (V = 0.104)
No	425 (78.8)	19 (70.4)	211 (84.4)	195 (74.4)		
Missing	19 (3.5)	4 (14.8)	4 (1.6)	11 (4.2)		
Chronic health concerns						
yes	171 (31.7)	12 (44.4)	69 (27.6)	90 (34.4)	χ^2^ (2, N = 516) = 7.4; p = 0.025 (V = 0.120)	χ^2^ (1, N = 493) = 3.5; p = 0.067
no	345 (64.0)	11 (40.7)	175 (70.0)	159 (60.7)		
missing	23 (4.3)	4 (14.8)	6 (2.4)	13 (5.0)		
Medication besides hormones						
yes	227 (42.1)	11 (40.7)	99 (39.6)	117 (44.7)	χ^2^ (2, N = 517) = 2.3; p = 0.313	χ^2^ (1, N = 494) = 2.2; p = 0.148
no	290 (53.8)	12 (44.4)	146 (58.4)	132 (50.4)		
missing	22 (4.1)	4 (14.8)	5 (2.0)	12 (4.6)		
Hospitalization for mental health concerns						
yes	69 (12.8)	4 (14.8)	30 (12.0)	35 (13.4)	χ^2^ (2, N = 518) = 0.7; p = 0.715	χ^2^ (1, N = 495)= 0.3; p = 0.596
no	449 (83.3)	19 (70.4)	215 (86.0)	215 (82.1)		
missing	21 (3.9)	4 (14.)	5 (2.0)	12 (4.6)		

^a^no transgender care pathway.

^b^traditional transgender care pathway.

^c^individual transgender care pathway.

^d^Kruskal-Wallis-test used as non-parametric test.

^e^Mann-Whitney U-test used as non-parametric test.

Due to the small number of participants in the NCP group, calculations were repeated, comparing only the ICP and the TCP group. Comparing the ICP group against the TCP group, participants from the ICP group were more likely to have an income around the poverty threshold, whereas participants from the TCP group were more likely to have an income above the poverty threshold (χ2 (2, N = 495) = 11.3; p = 0.001). Individuals with non-binary gender identity were more frequent in the ICP group (χ2 (1, N = 512) = 13.4; p = 0.000). Also, the ICP group reported significantly lower treatment satisfaction (z = −3.47, p = 0.001; Table 1). No relevant differences in demographics were discovered between the three research sites. Participants from Belgium reported a higher number of traditional treatment pathways (see supplementary material).

A logistic regression model (Table 2) found that non-binary gender identity and treatment satisfaction significantly predicted group membership. Participants with a non-binary gender were 6.7 times more likely to belong to the ICP group. Participants with higher treatment satisfaction were less likely to belong to the ICP group (OR: 0.6). The model showed a small effect (f^2^ = 0.11). Collinearity diagnostics did not show significant multicollinearity concerns for any of the variables in the model.

**Table 2:** Logistic regression analysis to predict the belonging to one of the care pathways.

	b ± SE	CI^a^	p	OR (95% CI)
Demographics				
Average income	−0.1 ± 0.1	−0.4–0.1	0.412	0.9 (0.7, 1.2)
Non-binary gender	1.9 ± 0.6	0.9–21.3	0.001	6.7 (1.9, 23.4)
Treatment-related predictors				
Treatment satisfaction	−0.5 ± 0.2	−0.9 – −0.3	0.001	0.6 (0.4, 0.8)
Constant	2.4 ± 0.7	1.1–3.8	0.001	11.1

R^2^ = 0.017 (Hosmer & Lemeshow) 0.073 (Cox & Snell), 0.098 (Nagelkerke). Model χ^2^ (6) = 30.153, p = 0.000.

^a^confidence intervals bootstrapped based on 5000 samples.

Four subgroups were detected within the ICP group and are summarized in Table 3.

**Table 3:** Subgroup analysis of individual transgender care pathways.

	n (%)	Commentary
Individual transgender care pathway	262 (100.0)	
only hormones	47 (17.9)	39 MAAB^a^, 8 FAAB^b^, 7 non-binary
hormones, top surgery	44 (16.8)	17 MAAB, 27 FAAB, 9 non-binary
hormones, top surgery, internal genital organs	64 (24.4)	64 FAAB, 9 non-binary
Hormones, Top surgery, external genital organs	1 (0.1)	
hormones, internal genital organs, external genital organs	101 (38.5)	101 MAAB, 4 non-binary
only top surgery	1 (0.1)	
top surgery, internal genital organs	1 (0.1)	
top surgery, internal genital organs, external genital organs	2 (0.1)	
internal genital organs, external genital organs	1 (0.1)	

^a^MAAB: Male assigned at birth.

^b^FAAB: Female assigned at birth.

## Discussion

In the present study, we explored demographic and clinical factors associated with individual and traditional transgender care pathways in a large multinational clinical sample of TGD individuals. Participants from Belgium reported a higher number of traditional treatment pathways. However, it is important to notice that the Belgian research site is also the one that has the longest experience with phalloplasty, which might cause individuals to consider this procedure more easily and inform themselves about it. How specific procedures offered by a center/country shape how people design their care path should be a main topic of future research as we still know very little about this potential interaction. Participants with a non-binary gender were 6.7 times more likely to undergo an individual transgender care pathway. This result is in line with previous research, reporting that non-binary TGD individuals are less likely to undergo transition-related medical interventions, especially gender-affirming genital surgery [15, 16, 29]. Prior research found that the primary sex anatomy, especially the presence or absence of a penis, contributes significantly to a distinct allocation as male or female [30]. As non-binary people usually do not follow a distinct and persistent gender allocation [1], it seems understandable that they also reject surgical procedures associated with such an assignment. Moreover, lower treatment satisfaction was associated with following an individual transgender care pathway. Lower satisfaction with previous transition-related interventions might have contributed to rejecting further interventions, even though a person had a wish for these interventions in the first place. However, the association between the continuation or discontinuation of transition-related interventions and variations in treatment satisfaction has not been investigated so far. Prior research mainly found structural circumstances (affordability, fear of discrimination from healthcare providers) as reasons for an interruption or discontinuation of transition-related interventions [29, 31–34]. Moreover, it must be noted that overall satisfaction with transition-related interventions was high in both groups (4.42 and 4.13).

A subgroup analysis of participants of the ICP group found several main individual transgender care pathways (Table 3). Around 20% of participants took hormones but did not undergo any surgery or have any plans to do so. Another 16.8% took hormones and underwent top surgery but did not plan to undergo gender-affirming genital surgery. Due to the distinctive physical effects that sex hormones can have on breast development [35] or voice pitch [36], both these groups could already have experienced a sufficient decrease of gender dysphoria and an increase in quality of life by these interventions. Limited genital dysphoria [13], fear of complications, and unsatisfactory aesthetic results must be considered as further reasons [37]. However, as our study did not provide sufficient data to support this interpretation, additional in-depth research is necessary in the future. Nearly a quarter of our participants – all assigned female at birth - followed an individual care pathway by accessing hormone therapy, top surgery, surgery of internal genital organs (hysterectomy, oophorectomy, salpingectomy), but no surgery of external genital organs (metoidioplasty, phalloplasty). This is in line with prior research reporting that TGD individuals assigned female at birth are less likely to undergo gender-affirming genital surgery than those assigned male at birth [13, 38]. The high number of complications and poor aesthetic results, especially with phalloplasty [37], need to be considered as reasons why participants in this group did not want to undergo gender-affirming surgery of the external genital organs. Moreover, the definite absence of menstruation after hysterectomy and oophorectomy might also have contributed to a significant lowering of gender dysphoria and an increase in quality of life, thus rendering further interventions unnecessary. Another group following an individual care pathway – all assigned male at birth- reported that they underwent hormone treatment, surgery of the internal (orchiectomy), and the external genital organs (vaginoplasty, penectomy; 38.5%). However, they did not undergo breast augmentation. From a clinical perspective, it must be noted that this subgroup might overlap with the group following a ‘traditional’ care pathway. Hormone therapy might have contributed to sufficient breast development [35], which is why these participants would not undergo any chest surgery, but they might have accessed the procedure if hormone therapy had not led to satisfactory breast development. In that case, they would have been classified as following a ‘traditional’ care pathway according to our grouping. However, when moving this subgroup into the TCP group and rerunning our analyses, we did not find significant changes in our results.

Finally, our study investigated a relatively small group of 27 TGD individuals who did not want to undergo any transition-related interventions. These participants might have primarily accessed care for psychological support or to evaluate their need for gender-affirming care. A high number of participants in this group reported a non-binary gender. This is in line with previous research investigating TGD individuals not seeking transition-related interventions [17]. As it has been reported repeatedly that non-binary individuals do undergo transition-related medical interventions less frequently [13, 15, 16], it is also likely that some of them do not want to undergo any treatment at all. In line with that, it has been found that some TGD individuals report that they do not experience distress associated with gender incongruence and, therefore, see no necessity for medical interventions [17]. Moreover, TGD individuals not seeking medical interventions might already experience a significant decrease in distress and an increase in quality of life by disclosing their feelings to loved ones or close friends and/or by using non-medical supplies (e.g., clothing, wigs, chest binder). However, it was noticeable that these participants reported significantly lower certainty about their gender than the other two groups (ICP, TCP). Regarding certainty about one’s gender, it could be assumed that some TGD individuals not seeking transition-related interventions are still in the phase of discovering their gender and, therefore, are unsure if they want to undergo transition-related medical interventions in the future. This struggle with questioning their gender and bodily appearance could have led to lower certainty about their gender.

### Limitations

Our regression model only had a small effect (f2 = 0.11) and explained a small amount of variance (Table 2). Using a regression model, we only investigated one direction of the relationship between the variables (Predictor-Outcome). However, it can be argued that the variables used in our models might interact with each other in both directions. Therefore, our data needs to be interpreted carefully, and no absolute conclusions can be drawn from it for clinical practice. Moreover, our study does not provide a detailed look at the variance for a certain surgical procedure, e.g., people undergoing hysterectomy and salpingectomy, but not oophorectomy. Also, all the clinics were in metropolitan areas, so TGD individuals from rural areas could have been excluded [39]. Healthcare systems were, to some degree, comparable in all three countries [40]. With some exceptions (e.g., breast augmentation in the Netherlands), coverage for most gender-affirming healthcare services was available in all three countries, too [41]. The assessment period was fixed and not related to the time of an individual finalization of treatment. This means that TGD individuals were invited to participate in a follow-up at a fixed point in time, irrespective of their individual need to further continue treatment or not. Therefore, some of the treatment pathways may not have been “finalized”. The classification of transgender care pathways into distinct groups only insufficiently represents the actual diversity of treatment requests of TGD individuals. Also, it does not incorporate any information on social gender affirmation, which could have an important influence on which transition-related interventions a person wants to undergo. It is also important to notice that, even though non-binary TGD individuals were more likely to undergo an ICP, approximately half of the participants identifying with binary gender wanted to undergo an ICP, too. Therefore, choosing an individual pathway of care is not exclusively associated with identifying as non-binary. Finally, as participants were recruited at specialized clinics rather than as a community sample, individuals who are not interested in gender-affirming care can be expected to be underrepresented.

## Conclusion

In conclusion, the present study highlights the various factors influencing transgender care pathways and highlights the complexity of individual gender-affirming care. With the goal of improving treatment satisfaction for TGD individuals, future research should focus on understanding how structural circumstances intersect with individual treatment preferences and influence transgender care pathways.

## Supplementary information

Supplementary Material

## Data Availability

We will consider sharing de-identified, individual participant-level data that underline the results reported in this article on receipt of a request detailing the study hypothesis and statistical analysis plan. All requests should be sent to the corresponding author. The corresponding author and lead investigators of this study will discuss all requests and make decisions about whether data sharing is appropriate based on the scientific rigor of the proposal. All applicants will be asked to sign a data access agreement.
