# Supplementary material for: Traditional and individual care pathways in gender-affirming healthcare for transgender and gender-diverse individuals - results from the ENIGI follow-up study
Source: Int J Impot Res. 2025 May 8;38(1):37–43. doi: 10.1038/s41443-025-01085-8 (PMC12864032; doi:10.1038/s41443-025-01085-8)
Supplement: Supplementary file 1 — Supplementary Material [file 41443_2025_1085_MOESM1_ESM.docx]

**Detailed Procedures**

Biographical data (e.g., age, educational level, marital status) and transgender-related information (e.g., gender) were assessed by a self-developed questionnaire. Participants were asked to give information on their age, which has been checked against the clinical record. Depending on the country, participants were asked to indicate their highest education completed. Subsequently, these answers were categorized as low, middle, and higher education. Answers on current employment (e.g., full-time, part-time, unemployed) were subsequently categorized as employed/unemployed. Participants indicated their average income compared to the poverty threshold of their country (below, around, above). The sex assigned at birth was assessed by the clinical records. Participants were asked to indicate their gender (male, female, trans woman, trans men, in between, other) and specify their answer by free text if necessary. Afterwards, we categorized gender as binary (e.g., trans woman) or non-binary (e.g., in between). Certainty about one's gender was assessed by a 5-point Likert scale (very uncertain, fairly uncertain, medium, fairly certain, very certain). Regarding treatment experiences, participants were asked if they had received transition-related medical interventions. For participants who answered "yes," subsequent questions explored whether they received hormonal treatment, if they had undergone any surgical treatment (e.g., gender-affirming genital surgery, facial surgery, vocal cord surgery), and if they had further treatment (e.g., hair removal). Due to inconsistencies and missing information in the self-report data, information on transition-related interventions were also retrieved from clinical records. Inconsistencies were resolved by checking the clinical records. All participants were asked if they intended to seek further transition-related interventions. Participants who answered "yes" were then asked to indicate which treatment they might seek (e.g., hormonal treatment, surgical treatment, further treatment).

Based on the treatment data, we constructed the primary outcome: following a traditional transgender care pathway, following an individual transgender care pathway, or not seeking transition-related medical interventions. Treatment satisfaction was assessed using a 5-point Likert scale (very unsatisfied, fairly unsatisfied, medium satisfied, fairly satisfied, very satisfied) for each transition-related intervention. If complications occurred, participants were asked to specify these. Satisfaction with physical appearance was measured using a 5-point Likert scale (very satisfied, fairly satisfied, medium, fairly unsatisfied, very unsatisfied). Traumatic events, chronic health concerns, medication besides hormones, and hospitalization for mental health concerns were asked using yes-no questions. For details on further measures used in the ENIGI follow-up study, see (Van De Grift et al., 2017). Moreover, the final survey can be provided upon request.

**Comparisons between countries**

Comparisons between the countries are reported in the following table. Participants differed regarding their age, education, employment, average income, sex assigned at birth, certainty about gender identity, hospitalization for mental health concerns, and treatment pathway. However, none of these differences reached a large effect size. From the clinical point of view, it appears unlikely that the differences had a major influence on the primary outcome of interest of the present study.

|  | **Belgium** | **Germany** | **Netherlands** | **Statistics** |
| --- | --- | --- | --- | --- |
| N (%) | 144 | 92 | 276 |  |
|  |  |  |  |  |
| Age (Mdn) | 35.00 | 33.00 | 39.00 | H=7.765; p=.021 |
|  |  |  |  |  |
| Education |  |  |  |  |
| Low | 12 | 12 | 22 | χ^2^ (2, N=520)= 18.046 ; p=.001 |
| Middle | 56 | 60 | 139 |  |
| High | 76 | 25 | 118 |  |
|  |  |  |  |  |
| Employment |  |  |  |  |
| Employed | 113 | 73 | 157 | χ^2^ (2, N=484)= 30.074 ; p=.001 |
| not employed | 22 | 19 | 100 |  |
|  |  |  |  |  |
| Average income (regarding poverty threshold) |  |  |  |  |
| Below | 16 | 29 | 53 | χ^2^ (2, N=518)=27.800; p=.001 |
| Around | 19 | 21 | 74 |  |
| Above | 108 | 47 | 151 |  |
|  |  |  |  |  |
| Sex assigned at birth |  |  |  |  |
| Male | 94 | 43 | 185 | χ^2^ (2, N=539)=11.680; p=.003 |
| Female | 55 | 54 | 108 |  |
|  |  |  |  |  |
| Non-binary gender |  |  |  |  |
| Yes | 135 | 87 | 271 | χ^2^ (2, N=539)=.928; p=.620 |
| No | 14 | 10 | 22 |  |
|  |  |  |  |  |
| Certainty about gender (Mean, Mdn) | 1.0 | 1.0 | 1.0 | H=6.823; p=.033 |
|  |  |  |  |  |
| Treatment satisfaction (Mdn) | 4.5 | 4.5 | 4.3 | H=1.126; p=.570 |
|  |  |  |  |  |
| No. of medical complications (Mdn) | 0 | 0 | 0 | H=2.402; p=.301 |
|  |  |  |  |  |
| Satisfaction with physical appearance (Mean, Mdn) | 2.0 | 2.0 | 2.0 | H=2.663; p=.264 |
|  |  |  |  |  |
| Traumatic events |  |  |  |  |
| Yes | 28 | 21 | 46 | χ^2^ (2, N=520)= 1.469 ; p=.479 |
| No | 116 | 76 | 233 |  |
|  |  |  |  |  |
| Chronic health concerns |  |  |  |  |
| yes | 50 | 27 | 94 | χ^2^ (2, N=516)= 1.384 ; p=.505 |
| no | 93 | 69 | 183 |  |
|  |  |  |  |  |
| Medication besides hormones |  |  |  |  |
| yes | 57 | 40 | 130 | χ^2^ (2, N=517)= 2.019 ; p=.364 |
| no | 85 | 57 | 148 |  |
|  |  |  |  |  |
| Hospitalization for mental health concerns |  |  |  |  |
| yes | 18 | 23 | 28 | χ^2^ (2, N=518)= .11.729 ; p=.003 |
| no | 124 | 73 | 251 |  |
|  |  |  |  |  |
| Treatment pathway |  |  |  |  |
| No treatment | 5 | 5 | 17 |  |
| Traditional | 96 | 39 | 115 |  |
| Individual | 48 | 53 | 161 |  |
